# Chitosan-ImH@γ-CD: a pH-sensitive smart bio-coating to enhance the corrosion resistance of magnesium alloys in bio-implants

**DOI:** 10.1039/d4ra04744c

**Published:** 2024-10-21

**Authors:** Sara Dehghan-Chenar, Hamid Reza Zare, Zahra Mohammadpour

**Affiliations:** a Department of Chemistry, Yazd University Yazd 89195-741 Iran hrzare@yazd.ac.ir

## Abstract

Magnesium alloys hold promise as bio-implants but are hindered by poor corrosion resistance. To overcome this, a pH-sensitive smart anti-corrosion bio-coating was developed using a layer-by-layer technique. The first layer consists of Imidazol@waterproofed γ-Cyclodextrin metal organic framework (ImH@waterproofed γ-CD MOF), which encapsulates ImH, a green inhibitor, in waterproofed γ-CD MOF. The second layer is composed of 1% w/v chitosan. ImH@waterproofed γ-CD MOF was characterized by SEM, FTIR, and XRD. The corrosion parameters of the smart bio-coating were investigated through potentiodynamic polarization (Tafel) plots and electrochemical impedance spectroscopy (EIS) in simulated body fluid (SBF). The results indicate that when the magnesium alloy coated with the chitosan-ImH@γ-CD composite is placed in the SBF solution, the pH near the corrosion site increases over time. This increase in pH leads to the release of imidazole as a corrosion inhibitor, effectively preventing surface corrosion by forming a protective layer on the alloy's surface. The chitosan-ImH@γ-CD composite exhibits an inhibition efficiency of 97.27% after 5 days of immersion in SBF. Additionally, the cell viability on the chitosan-ImH@γ-CD composite surface is significantly higher than on uncoated Mg alloy, promoting MC3T3-E1 cell proliferation. Alkaline phosphatase results also indicate improved differentiation of MC3T3-E1 cells with the chitosan-ImH@γ-CD composite.

## Abstract 2

Fabrication of chitosan-imidazol@γ-cyclodextrin metal–organic framework composite as a pH-sensitive smart anti-corrosion bio-coating and investigation of its corrosion parameters in simulated body fluid corrosive solution.

## Graphical abstract

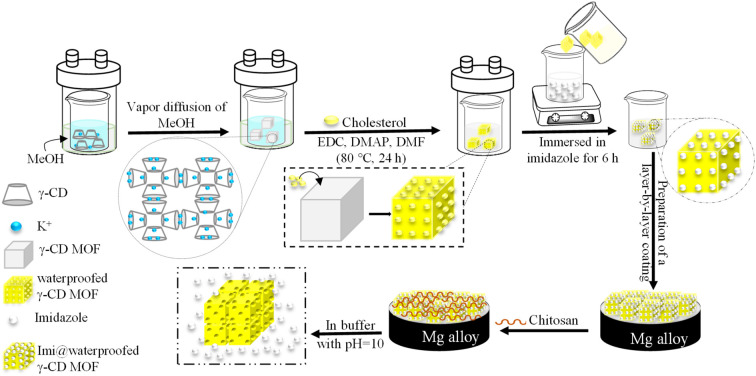

## 1. Introduction

Magnesium alloys have numerous biomedical applications due to their high biocompatibility.^1^ Magnesium plays a vital role in metabolic reactions and biological mechanisms. Given the abundant amount of magnesium ions in the human body, magnesium alloys can be utilized as bio-implants.^2^ Since magnesium bio-implants are degradable, secondary surgeries are not required, making them appealing to scientists.^3^ However, the limited corrosion resistance of magnesium metal restricts its applications.^4^ Corrosion control can be achieved through the development of new alloys,^2^ the use of coatings,^5^ and surface modification.^6^ In a study by Yin *et al.*^7^ the effect of zinc content on the microstructure, mechanical properties, and corrosion behavior of Mg–Zn–Mn alloy in simulated body fluid (SBF) was investigated. It was found that 1 wt% zinc provides the best anti-corrosion properties, which is why Mg–Zn–Mn alloys are employed in biological studies.

The use of protective coatings is a widespread and cost-effective approach for corrosion protection. These coatings create a physical barrier that prevents the metal surface from coming into contact with the corrosive environment.^8^ The use of smart coatings can further enhance the efficiency of corrosion protection. A smart coating has the ability to improve its protective properties in response to environmental stimuli, such as changes in pH,^9^ temperature,^10^ and the concentration of aggressive ions.^11^ In smart corrosion inhibiting coatings, micro/nano capsules are utilized as carriers to enclose corrosion inhibitors.^12^ A change in pH can trigger the release of the corrosion inhibitor.^13^

While organic inhibitors are commonly used to reduce the corrosion rate of metals, many of them have toxic properties that contribute to environmental problems. Therefore, environmentally friendly green inhibitors are preferred.^14^ Imidazole and its derivatives are known as green inhibitors. Imidazole, which is an aromatic molecule, has been extensively studied for its corrosion inhibition performance.^15,16^ Mishra *et al.* conducted a comprehensive review of recent advancements in imidazole-based compounds as corrosion inhibitors for metals across various environments.^17^

Choosing a type of particle that can create a temporary bond with the inhibitors and break as soon as the corrosion by-products are released to lead the release of the inhibitor is one of the most important features for choosing micro/nano carrier capsules. Metal–Organic Frameworks (MOFs) display extraordinary properties, such as high surface areas, ultrahigh porosity, and thermal stability, which make them a potential candidate for this purpose.^18^ As a typical biocompatible MOF, cyclodextrin metal–organic frameworks (CD-MOFs) have recently received considerable attention due to their edible, renewable, and biodegradable nature.^19^ CD-MOFs are constructed from natural cyclodextrins and alkali metal salts. Cyclodextrins (CDs) are cyclic oligosaccharides that are enzymatically produced from starch. CDs exist in three forms, namely, α, β, and γ, which represent six, seven, or eight glucopyranose units, respectively. CDs can form host-guest inclusion complexes consisting of a hydrophobic internal cavity and a hydrophilic external surface allowing them to combine with many inorganic or organic molecules.^20^ Research indicates that γ-CD generally demonstrates lower cytotoxicity in comparison to β-CD.^21^ The larger cavity size of γ-CD, comprising eight glucose units, enables it to accommodate larger or bulkier guest molecules than α-CD and β-CD. This characteristic is particularly significant for the formation of metal–organic frameworks, which can be advantageous in drug delivery systems and various other applications.^21,22^ γ-CD MOF shows promising applications in biomedicine, pharmaceutics and other fields. However, these application face serious challenges due to the poor water stability and rapid disintegration of γ-CD MOFs.^23^ So far, several strategies have been reported to improve the stability of γ-CD MOFs in water. Singh *et al.* developed a facile and one step-method to enhance the water stability of γ-CD MOF nanoparticles through surface modification with cholesterol.^24^

In 2015, Liu *et al.* conducted an investigation into the corrosion inhibition of carbon steel in 0.5 M hydrochloric acid solutions using β-cyclodextrin-modified natural chitosan.^25^ The findings indicated that β-CD-chitosan functions as an effective inhibitor for carbon steel corrosion in hydrochloric acid solutions, with its inhibition efficiency enhancing as the concentration increases.^25^ In 2021, Dehghani *et al.* developed a silane-based composite film incorporating β-CD-benzimidazole macromolecules.^26^ The results revealed that the introduction of the inhibitive complexes led to an increase in the resistance of immersed steel in 3.5% NaCl after 5 hours, demonstrating an inhibition performance of 84%.

In this study, the layer-by-layer technique was used to create a smart coating on the surface of a magnesium alloy. Initially, the first layer consisted γ-CD MOF containing an imidazole corrosion inhibitor was placed on the electrode surface. Next, the second layer, consisting of chitosan, was placed on the first layer. Experimental data indicate that the corrosion process is initiated, followed by a change in the pH of the corrosive site. This leads to the release of the imidazole inhibitor, resulting in a reduction in the corrosion rate. The inhibitor loading capacity and the amount of inhibitor released at different pHs were investigated using UV-vis analysis. The results demonstrate that the ImH@γ-CD MOF smart coating, with pH-stimulus responsive, can effectively decrease the corrosion of the magnesium alloy in the SBF corrosive environment.

## 2. Experimental methods

### 2.1. Materials

Gamma cyclodextrin (γ-CD, ≥98%) and chitosan (medium molecular weight) were purchased from Sigma-Aldrich Company. Potassium hydroxide (KOH, ≥85%), cholesterol (CHS, ≥95%), 4-dimethylamino pyridine (DMAP, ≥99%), 1-ethyl-3-3-dimethylaminopropylcarbodiimide (EDC, ≥98%), and imidazole (C_3_H_4_N_2_, ≥99%) (ImH) were provided by Merck Company. The solvents used were methanol (MeOH), ethanol (EtOH), *N*,*N*-dimethylformamide (DMF), and acetic acid (CH_3_COOH) and were used as received without purification.

### 2.2. Instruments and methods

The crystal structures of γ-CD MOF, waterproofed γ-CD MOF, and ImH@waterproofed γ-CD MOF powders were determined using X-ray diffraction (XRD) measurements. An Asenware AWDX300 XRD diffractometer, equipped with a Cu Kα radiation source with *λ* = 0.154 nm, was used in the 2*θ* range from 5° to 80° with a step size of 0.05°. Cross-sectional and surface views of the produced coatings were studied using a scanning electron microscope (SEM) equipped with an energy dispersive X-ray spectroscopy (EDS) microanalyzer, specifically a Philips XL-30 microscope. The average size of the synthesized MOFs was estimated using Digimizer software. The γ-CD MOF, waterproofed γ-CD MOF, ImH and ImH@waterproofed γ-CD MOF were characterized using Fourier transform infrared spectroscopy (FTIR, Bruker, Vector 22, Germany) in the range of 400–4000 cm^−1^ using KBr pellets. The loading and releasing profile of imidazole were calculated using UV-vis absorption (PHYSTEC Spectrometer UVS-2500, Iran) at *λ*_max_ = 208 nm. Electrochemical experiments were performed using an Ivium potentiostat/galvanostat (Ivium, Vertex One, Netherlands) equipped with Ivium software. A saturated calomel electrode (SCE), a platinum rod and a magnesium alloy (Mg-1%–Zn-1.2%–Mn alloy) cylinder were used as the reference, counter, and working electrodes, respectively. Before each measurement, the working electrode was immersed in simulated body fluid (SBF), and an open-circuit potential (*E*_OCP_) was applied for 4000 s to stabilize the OCP value of the electrode. Potentiodynamic polarization curves were recorded at a potential scan rate of 1 mV s^−1^ and ± 200 mV *versus* E_OCP_ at room temperature. The corrosion potential (*E*_corr_) and corrosion current density (*J*_corr_) were obtained by extrapolating the cathodic and anodic regions of the Tafel plots. The Electrochemical impedance spectroscopy measurements (EIS) were recorded within the frequency range of 10 mHz to 100 kHz with a 10 mV amplitude at open-circuit potential (*E*_OCP_). The obtained impedance data were simulated using ZsimpWin software. A Faraday cage was used to remove noise in all electrochemical experiments.

### 2.3. Synthesis of γ-CD MOF

Crystals of γ-CD MOF were synthesized using a vapor diffusion method described in the literature.^27^ In this method, the crystal structures of γ-CD MOF grow in the diffused vapor of MeOH. To begin, 1.3 g of γ-CD and 0.4 g of KOH were dissolved in 20.0 mL of deionized water and stirred for 5 minutes at 25 °C. The mixture was then transferred into a Teflon autoclave containing methanol and placed in an oven at 80 °C for 15 h to allow for the formation of crystalline particles through the vapor diffusion of MeOH. The resulting product was filtered using filter paper and washed with MeOH before being dried in an oven at 40 °C for 6 h.

### 2.4. Increasing the water stability of γ-CD MOF

According to a method reported in the literature^24^ with some modifications, it is possible to increase the water stability of γ-CD MOF by attaching cholesterol to its structure. 100.0 mg of γ-CD MOF, 13.0 mg of cholesterol, 7.6 mg EDC, and 3.2 mg DMAP were added to 3.0 mL of DMF and stirred for 30 minutes. After transferring to a Teflon autoclave, it was placed in an oven at 80 °C for 24 h, and the resulting product was centrifuged and dried at room temperature for 24 h.

### 2.5. Loading of corrosion inhibitor into γ-CD MOF

To load imidazole as a corrosion inhibitor into waterproofed γ-CD MOF, 30.0 mg of the waterproofed γ-CD MOF was added to 2.0 mL of a solution of 1000.0 ppm imidazole in water and stirred for 6 h, then centrifuged. The washed MOFs with water were dried at room temperature. Finally, the loading capacity was analyzed by UV-vis is absorption spectra.

### 2.6. Preparation of a layer-by-layer coating

To prepare a layer-by-layer coating, magnesium alloy (Mg-1%–Zn-1.2%–Mn) electrodes were polished sequentially with 600, 1200, and 2500 grit sandpapers, cleaned in acetone under ultrasonic conditions for 5 minutes, washed with deionized water and then dried. The first layer contains 8.0 μL of a 5.0 mg mL^−1^ solution of waterproofed γ-CD MOF loaded with imidazole dispersed in an ethanol solution, which is dripped onto the surface of the electrode. After drying, another drop is dripped to completely cover the surface of the electrode. The second layer contains 8.0 μL of a 1.0% w/v chitosan solution dissolved in 1.0% v/v acetic acid, which is placed on the electrode using the same method.

### 2.7. Release study of corrosion inhibitor from layer-by-layer coating

To calculate the inhibitor release rate from the layer-by-layer coating at different pH values, the electrode containing the layer-by-layer coating was placed in buffer solutions with pH values of 3.0, 7.0, and 10.0. The solution was kept at 25 °C. After 5.0, 10.0, 15.0, 20.0, 30.0, 45.0 and 60.0 minutes, the percentage of the released inhibitor was calculated using the absorption at *λ*_max_ of the ImH spectrum.

### 2.8. MTT test assay

The MTT assay is a commonly used method to measure cell viability and proliferation. The substance used for the MTT test was a 3-(4,5-dimethylthiazol 2-yl)-2, 5-diphenyltetrazolium salt, which convert into a blue formazan product due to viable mitochondria in active cells. The amount of formazan formed is proportional to the number of viable cells. Absorbance of the solubilized formazan at a specific wavelength (usually around 570 nm) was measured using a microplate reader. Absorbance values were compared between treated and untreated cells. Higher absorbance indicates more viable cells, while a decrease suggests reduced viability. Cell studies were performed with MC3T3-E1 cells. MC3T3-E1 cells were seeded at a density of 2 × 10^4^ cells per well and incubated for 24 hours to allow optimal attachment. After that, the medium was replaced with 250 μL of coated and uncoated alloy supernatant. Additionally, cultured cells in DMEM medium containing 10% FBS and no extract were considered as the control group. After 1, 3, and 7 days, the culture medium was removed, and a solution of 25 μL MTT dissolved in PBS was added to the cells. The samples were incubated with MTT in DMEM for 4 hours at 37 °C, 5% CO_2_. Then, 250 μL DMSO solution was added to each well, and 100 μL of the supernatant was analyzed by a microplate reader (Microplate reader, Awareness, Stat-Fax 2100, America) at 570 nm in 96-well plates.

### 2.9. ALP activity assay

Alkaline phosphatase was used to evaluate the differentiation level of MC3T3-E1 cells. The protocol for converting *p*-nitrophenyl phosphate (*p*-NPP) to *p*-nitrophenol was used to assess ALP activity at specified time points. Similar to the MTT test, MC3T3-E1 cells were seeded at a density of 4 × 10^4^ cells per well and incubated for 24 hours to allow optimal attachment. After that, the medium was replaced with 250 μL of coated and uncoated alloy supernatant. Additionally, cultured cells in DMEM medium containing 10% FBS and no extract were considered as the control group. After 7 and 14 days, the cells were lysed in a solution of Triton X-100 and 1× assay buffer. After centrifugation, the cell layer was suspended before removing the lysis solution. The lysed cells were then transferred to 96-well plates, and 50 μL of a colorimetric alkaline phosphatase substrate was added to each well. The plate was shaken for 60 minutes at 37 °C in a 5% CO_2_ humidified atmosphere, and absorbance readings were taken using a microplate reader (Microplate reader, Awareness, Stat-Fax 2100, America). ALP activity was determined by the amount of *p*-nitrophenol (p-NP) produced during the hydrolysis of *p*-nitrophenyl phosphate (*p*-NPP) in the presence of ALP as a catalyst.

## 3. Results and discussion

### 3.1. Characterization of γ-CD MOF, waterproofed γ-CD MOF, and ImH@γ-CD MOF

**Fig. 1:**
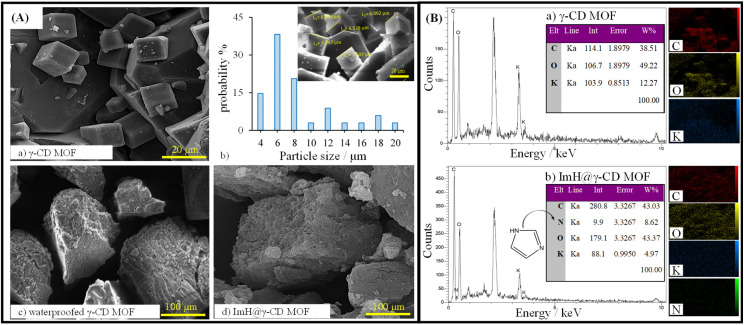
(A) Scanning electron microscope (SEM) images of (a) γ-CD MOF, (b) size distribution of γ-CD MOF, (c) waterproofed γ-CD MOF, and (d) ImH@γ-CD MOF. (B) Energy dispersive X-ray spectroscopy (EDS) and elemental scattering map of (a) γ-CD MOF and (b) ImH@γ-CD MOF.

**Fig. 2:**
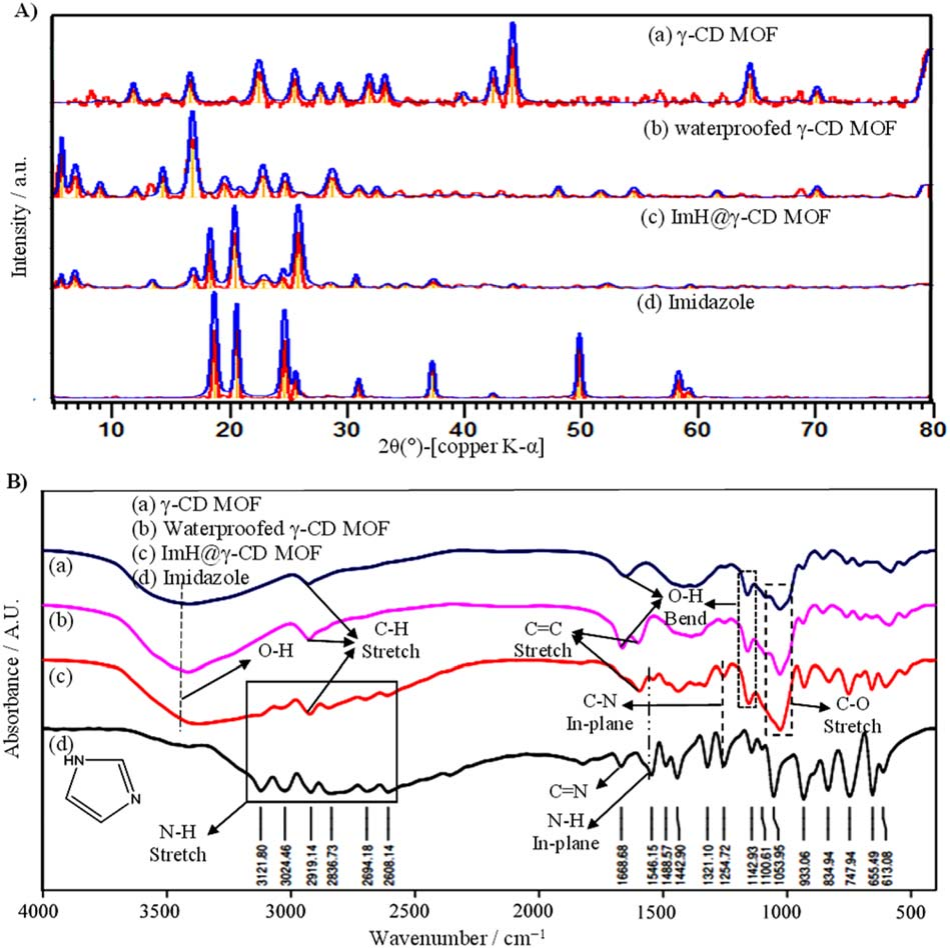
(A) X-ray diffraction (XRD) patterns and (B) FTIR spectra of (a) γ-CD MOF, (b) waterproofed γ-CD MOF, (c) ImH@γ-CD MOF, and (d) Imidazole.

SEM images obtained from the dried samples reveal intriguing microstructural properties that provide insight into the structural organization of all particles. Fig. 1A displays the SEM images of the synthesized γ-CD MOF, waterproofed γ-CD MOF, and Imidazole-loaded γ-CD MOF (ImH@γ-CD MOF). The SEM images depict cubic shapes for all γ-CD MOFs both before and after the inhibitor loading. A body-centered cubic crystal was constructed with six γ-CDs (*i.e.* γ-CD_6_), each connected to its adjacent unit (*i.e.* γ-CD) through symmetrical K^+^ connections. This arrangement results in eight K^+^ ions surrounding each body-centered cubic crystal, creating a cohesive 3D structure.^23^ The cubic crystal structure of γ-CD MOF exhibited a size distribution ranging from 4 to 20 μm, determined through image analysis using Digimizer software (Fig. 1A(b)). Fig. 1B illustrates the chemical composition of both γ-CD MOF and ImH@γ-CD MOF, characterized by EDS. The EDS spectrum shows that the emission from carbon and oxygen atoms can be attributed to the organic linker of γ-CD, while the emission from potassium atoms originates from the metal source of γ-CD MOF. Upon loading the MOF with the ImH inhibitor, a distinct nitrogen emission peak becomes evident, corresponding to the corrosion inhibitor ImH with the chemical formula C_3_H_4_N_2_. The inset of each EDS spectrum provides the elemental percentage values. Furthermore, elemental mapping indicates a uniform distribution of C, K, O, and N atoms within the ImH@γ-CD MOF. This observation confirms the successful loading of ImH into the pores of γ-CD MOF. To validate the structure of the synthesized γ-CD MOF, a powder X-ray diffraction characterization of the particles was conducted. The XRD patterns of (a) γ-CD MOF, (b) waterproofed γ-CD MOF, (c) ImH@γ-CD MOF and (d) imidazole are presented in Fig. 2A. This outcome verifies the crystalline nature of γ-CD MOFs, as the diffraction peaks align with those reported in the existing literature for this structure.^28^ The characteristic diffraction peaks of γ-CD MOFs were observed at approximately 2*θ* = 13.2, 16.7, and 23.1, which are consistent with the literature.^29,30^

The additional peaks observed in the 2*θ* range of 10–25° for the waterproofed γ-CD MOF, compared to the γ-CD MOF, indicate the presence of cholesterol within the structure of the waterproofed γ-CD MOF.^31^ Furthermore, the intensity of the characteristic peaks of the MOF indicates the preservation of its structure. The diffractogram of imidazole shows sharp and prominent peaks within the 2*θ* range of 10–30°, which exactly matches the reference diffraction pattern (22-1759 in the ICDD database).^32^ Subsequently, the ImH@γ-CD MOFs exhibited distinct diffraction peaks of ImH after being loaded, suggesting the successful loading of ImH. The encapsulation of ImH in γ-CD MOF was also revealed using FTIR spectroscopy. The FTIR spectra of all the samples are given in the spectral range between 400 and 4000 cm^−1^ (Fig. 2B). The FTIR spectrum of (a) γ-CD MOF, (b) waterproofed γ-CD MOF, and (c) ImH@γ-CD MOF (Fig. 2B) display a wide absorption peak within the range of 3030–3670 cm^−1^, indicative of the stretching vibrations of OH groups within the glucose units. Additionally, a peak at 2927 cm^−1^ is observed, corresponding to the stretching vibrations of C–H bonds in both the CH_2_ and CH groups.^33^ Peaks at 1029 and 859 cm^−1^ are respectively related to C–O and C–C stretching vibrations in γ-CD MOF, waterproofed γ-CD MOF, and ImH@γ-CD MOF.^30^ Peaks at 1643 and 1158 cm^−1^ are attributed to OH group bending vibrations.^29^ Most of the peaks associated with γ-CD MOF are presented in the spectrum of ImH@γ-CD MOF. The emergence of certain new peaks in this spectrum can be ascribed to the encapsulation of the ImH corrosion inhibitor (Fig. 2B(d)). The peaks located at 1546 and 1254 cm^−1^ correspond to the in-plane N–H and in-plane C–N, respectively.^34^ The bonding of hydrogen to the N atoms of imidazole led to a series of weak peaks between 3200 and 2500 cm^−1^. So, the FTIR results generally demonstrate that imidazole molecules were successfully encapsulated in the γ-CD MOF.

### 3.2. Loading and pH-responsive release profile of the imidazole corrosion inhibitor

**Fig. 3:**
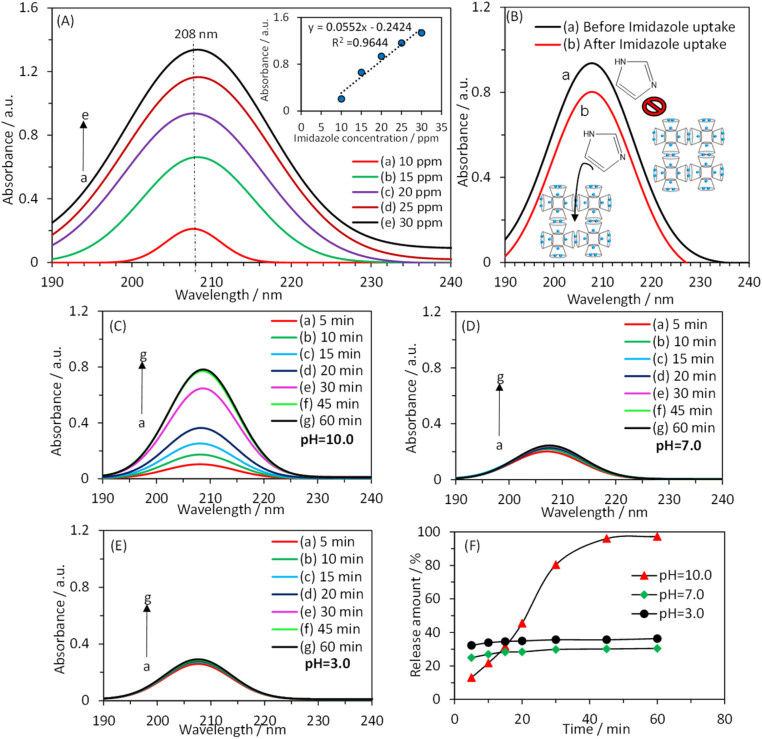
(A) UV-vis absorption spectra of different concentrations of imidazole (10.0, 15.0, 20.0, 25.0, and 30.0 ppm). Inset shows the absorbance changes related to *λ*_max_*versus* imidazole concentration. (B) UV-vis absorption spectra of a 1000.0 ppm imidazole solution (a) before and (b) after interaction with waterproofed γ-CD MOF particles. UV-vis absorption spectra of imidazole released after keeping the layer-by-layer coated electrode for 5.0, 10.0, 15.0, 20.0, 30.0, 45.0 and 60.0 min in 0.1 M phosphate buffer with pHs (C) 10.0, (D) 7.0 and (E) 3.0. (F) Variations in the percentage of imidazole released from waterproofed γ-CD MOF as a function of storage time in the buffer at three different pH.

Fig. 3A shows the UV-vis absorption spectrum of a solution containing imidazole at different concentrations. The inset of Fig. 3A demonstrates a linear relationship between the absorbance value at *λ*_max_ (*λ*_max_ = 208 nm) and the imidazole concentration. Fig. 3B presents the UV-vis absorption spectrum of a 1000.0 ppm imidazole solution in the absence and presence of waterproofed γ-CD MOF. The decrease in absorbance intensity in the presence of waterproofed γ-CD MOF indicates that the imidazole was encapsulated within the MOF framework. By measuring the absorbance at *λ*_max_ and using the calibration plot, the remaining imidazole concentration in the solution was determined after encapsulation into waterproofed γ-CD MOF. Consequently, the imidazole loading capacity within the waterproofed γ-CD MOF particles was calculated to be 37.3% using the following equation: loading capacity (wt%) = (imidazole uptake/(imidazole uptake + waterproofed γ-CD MOF)) × 100.

**Scheme 1:**
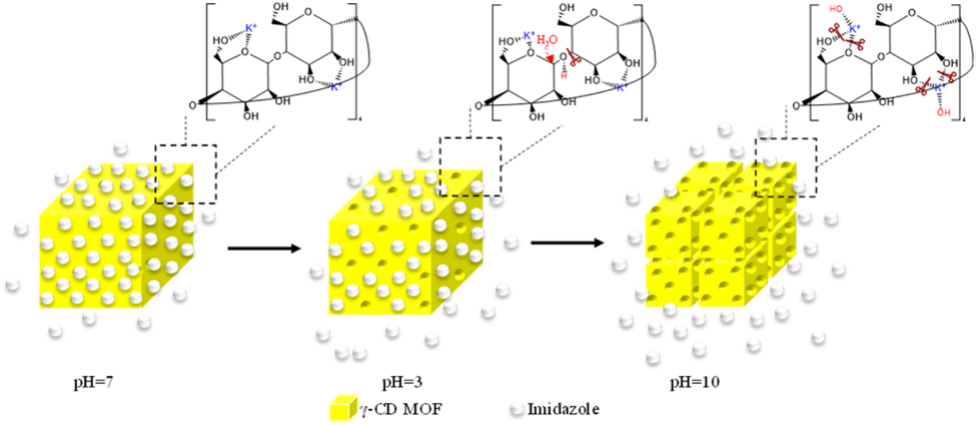
Schematic representation of the pH-responsive release of imidazole at different pH.

To investigate the impact of pH on the controlled release of imidazole encapsulated within the waterproofed γ-CD MOF, UV-vis absorption spectroscopy was used to analyze the release kinetics of imidazole. The experiments were conducted out in a 0.1 M phosphate buffer solution at three distinct pH levels: basic (pH 10.0), neutral (pH 7.0), and acidic (pH 3.0). The release profiles were measured at specified intervals of 5, 10, 15, 20, 30, 45, and 60 minutes in the buffer solutions being examined, as illustrated in Fig. 3C–E. Fig. 3F shows the time-dependent percentage of imidazole released from the waterproofed γ-CD MOF under these varying pH conditions. The data obtained indicate that, after 60 minutes, the percentages of imidazole released in basic, neutral, and acidic solutions are 97.2%, 30.4%, and 36.4%, respectively. In an acidic environment, the hydrolysis of the glycosidic bond is enhanced. Consequently, when immersed in an acidic solution, ImH@waterproofed γ-CD MOF undergoes gradual and partial ring opening of γ-CDs, leading to the degradation of the γ-CD MOF and accelerating the release of imidazole. The stability of γ-CD MOFs is generally compromised in humid and aqueous environments due to weak potassium–oxygen coordination, making them susceptible to attack by polar groups, particularly water molecules. Under basic conditions, the presence of more polar groups, such as hydroxide, is likely to initiate an attack on the metal centers. This process results in the cleavage of weak coordination bonds of potassium–oxygen, ultimately facilitating the release of imidazole^30^ as shown in Scheme 1.

### 3.3. Electrochemical assessment of corrosion behavior of magnesium alloy coated with various coatings

**Fig. 4:**
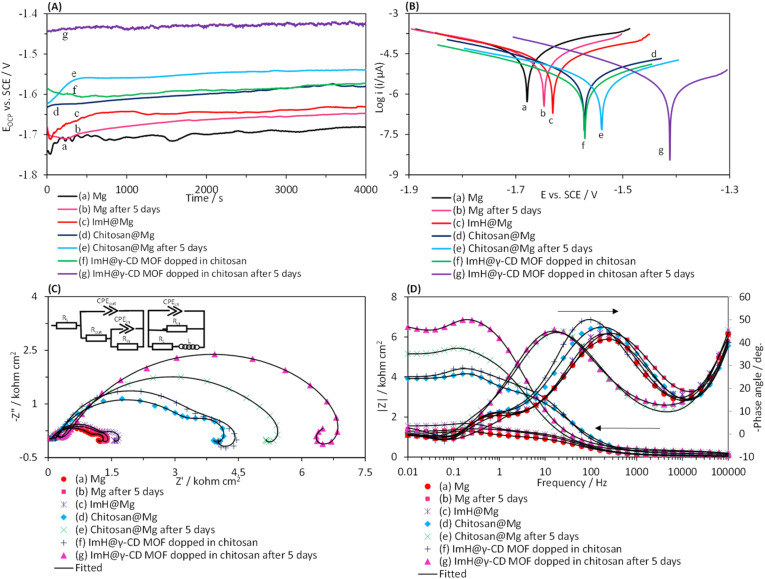
Plots of (A) open-circuit potential (*E*_OCP_) *versus* time, (B) potentiodynamic polarization, (C) Nyquist and (D) Bode and Bode-phase for (a) Mg alloy, (b) Mg alloy after 5 days immersed in SBF, (c) imidazole coating (ImH@Mg), (d) chitosan coating (chitosan@Mg), (e) chitosan coating after 5 days immersion in SBF, (f) imidazole loaded into the structure of γ-CD MOF doped in chitosan (composite of chitosan-ImH@γ-CD) and (g) composite of chitosan-ImH@γ-CD after 5 days immersion in the SBF corrosive solution at 25 °C.

In this section, we examined the corrosion phenomena of several coatings applied in droplet form on the surface of the magnesium alloy. The coatings studied were imidazole (ImH@Mg), chitosan (chitosan@Mg), and a composite of chitosan-ImH@γ-CD (imidazole loaded into the structure of γ-CD MOF doped in chitosan). The corrosion assessment was conducted in a simulated body fluid (SBF) solution. Fig. 4A and B present the time-dependent changes in open circuit potential (*E*_OCP_) and Tafel plots for the coated magnesium alloy electrodes, respectively. To understand the corrosion processes, it is derived the kinetic parameters from the anodic and cathodic regions of the Tafel plots. The results are summarized in Table 1, including the corrosion potential (*E*_corr_) corrosion current density (*J*_corr_) and corrosion rate (CR) which were calculated using this equation (CR = (0.13 × *J*_corr_ × EW)/*d*). Where EW and *d* stand for the equivalent weight and the density of the magnesium alloy substrate, respectively. Furthermore, this equation (IE% = (1 − (*J*_corr(*i*)_/*J*_corr(0)_)) × 100) was used to calculate the inhibitory efficiency percentage. In this equation, *J*_corr(*i*)_ and *J*_corr(0)_ signify the corrosion current density of the magnesium alloy coated with different coatings and the uncoated magnesium alloy, respectively. By examining the OCP plots, it was observed that when a coat is applied to the surface of the magnesium alloy, the potential shifts towards less negative values. The shift of OCP value towards the less negative side is more pronounced for the composite of chitosan-ImH@γ-CD compared to the coatings of chitosan@Mg, ImH@Mg, and Mg alloy. This shift in OCP suggests a decrease in the susceptibility of the alloy to the corrosion process, but the OCP values do not provide any information on the corrosion kinetics.^35^ A more negative OCP value for the Mg alloy indicates its active behavior, possibly due to oxidation into Mg^2+^ ions that change the equilibrium potential to a more negative value.^36^ The *E*_OCP_ values of the modified electrodes with coatings reach a constant value after exposure to the corrosive environment for 4000 s. This steady state condition represents the lack of variation in the interface between the substrate and the environment solution.^37^ As shown in Fig. 4A, with an increase in the immersion time of the magnesium alloy in the SBF solution, the OCP values gradually shift to less negative values and eventually reach a constant value. These results indicate the formation of a Mg(OH)_2_ layer on the Mg alloy.^38^ Therefore, in subsequent experiments, the electrodes were first placed in the corrosive environment for 4000 s until their open-circuit potential (*E*_OCP_) reached a constant value. The Tafel curves for Mg, ImH@Mg, chitosan@Mg and the composite of chitosan-ImH@γ-CD after reaching the steady state and after 5 days of immersion in SBF corrosive solution are depicted in Fig. 4B. The data in Table 1 show that the corrosion parameters of the magnesium alloy are affected by the nature of the coating and the duration of immersion in the SBF corrosive solution. According to curve c in Fig. 4B, the addition of imidazole shifts the corrosion potential to a less negative value and increases the anodic resistance.^39^ The imidazole inhibitor decreases the destruction caused by corrosion of the magnesium alloy by adsorption on the metal surface (curve c in Fig. 4B). Chitosan exhibits reasonably good anticorrosive activity due to its polymeric nature, providing excellent protection after adsorption onto the metal surface (curves d in Fig. 4B). Chitosan contains various polar substituents that not only enhance its solubility in solutions but also increase its ability to interact with the metal surface. Clearly, chitosan possesses numerous electron-rich polar sites or functional groups, including –OH (hydroxyl), –NH_2_ (amino), –CH_2_OH (hydroxymethyl), –O– (cyclic and acyclic ether), and –NHCOCH_3_ (amide), which allow for easy adsorption on metallic surfaces. Chitosan exhibits the ability for both physisorption and chemisorption.^40^ Using the composite of chitosan-ImH@γ-CD, the corrosion current density (*J*_corr_) reaches the lowest value (curve f in Fig. 4B). Therefore, the chitosan-ImH@γ-CD composite provides the highest corrosion protection for the magnesium alloy surface in the SBF media. The data in Table 1 show that the chitosan-ImH@γ-CD composite coating exhibits the lowest corrosion rate and the highest inhibition efficiency in the SBF solution. As the immersion time of the magnesium alloy in the SBF solution increases (after 5 days), the corrosion potential shifts towards less negative values. When the magnesium alloy is immersed in the SBF solution for 5 days, a layer of magnesium hydroxide (Mg(OH)_2_) forms on its surface, preventing corrosion.^41^ By placing the magnesium alloy coated with the chitosan-ImH@γ-CD composite in the SBF solution, the solution's pH increases near the corrosion site over time. This results in the release of imidazole as a corrosion inhibitor, which forms a protective layer on the surface of the alloy, thus protecting it against corrosion. The pH of the simulated body fluid (SBF) solution is maintained at 7.4. Upon immersion of the magnesium alloy in SBF, the pH of the solution increases from 7.4 to 10 over a period of five days. This change is attributed to the reaction between chloride ions (Cl^−^) present in the surrounding environment and Mg(OH)_2_, which results in the formation of highly soluble MgCl_2_ and hydroxide ions (OH^−^). Consequently, this reaction induces a significant increase in pH, accompanied by the production of Mg^2+^ and Cl^−^ ions within the reaction zone.^42^ Therefore, compared to the chitosan@Mg coating after 5 days of immersion in the corrosive solution, the chitosan-ImH@γ-CD composite coating exhibits the highest inhibition efficiency (97.27%), the lowest corrosion rate (1.38 mpy), and the minimum corrosion current density (3.00 μA cm^−2^) (Table 1).

**Table 1:** Kinetic and thermodynamic data obtained from the potentiodynamic polarization plots in Fig. 4B, corresponding to the Mg alloy, ImH@Mg, chitosan@Mg and chitosan-ImH@γ-CD composite before and after immersion in SBF solution for 5 days at 25 °C

Parameters	Sample						
	Before immersion in SBF solution for 5 days				After immersion in SBF solution for 5 days		
	Mg	ImH@Mg	Chitosan@Mg	Composite of chitosan-ImH@γ-CD	Mg	Chitosan@Mg	Composite of chitosan-ImH@γ-CD
*E*_corr_ (V)	−1.68	−1.63	−1.57	−1.57	−1.64	−1.54	−1.41
*I*_corr_ (μA)	22.06	14.20	3.32	1.77	19.90	1.62	0.60
*J*_corr_ (μA cm^−2^)	110.30	71.00	16.61	8.84	99.50	8.10	3.00
*β*_a_ (mV)	97	132	120	69	110	79	54
−*β*_c_ (mV)	121	128	92	75	125	81	38
CR (mpy)	50.89	32.76	7.66	4.08	45.90	3.73	1.38
IE%	0	35.63	84.94	91.98	9.79	92.65	97.27

To further investigate the corrosion phenomena and achieve effective corrosion inhibition using the coating prepared in the SBF solution, the EIS of all coatings were examined in the frequency range of 10 MHz to 100 kHz at *E*_OCP_. Nyquist, Bode, and Bode-phase plots for uncoated and coated Mg alloy are presented in Fig. 4C and D, respectively. The Nyquist plots reveal a small inductive loop with a center below the real axis (at positive values of Z′′) at low frequencies for all curves. The appearance of an inductive loop at low frequencies in the Nyquist plot is attributed to the adsorption process.^43^ An electrical equivalent circuit (EEC) model with three-time constants has been used to derive the kinetic parameters of the corrosion process from the EIS plots (Fig. 4C, inset). The equivalent circuit model consists *R*_s_, representing the resistance of the electrolyte solution; CPE_out_, indicating the capacity of the external layer; *R*_out_, representing the resistance of the external layer; CPE_in_, the capacity of the internal layer; *R*_in_, indicating the resistance of the internal layer, CPE_ct_, representing the charge transfer capacity and *R*_ct_ indicating the charge transfer resistance. *L* is the inductance, and *R*_L_ is the resistance of the inductor element. The rapid oxidation of the magnesium generates Mg^2+^ ions, leading to the formation of an additional protective layer through the adsorption of corrosion products onto the sample's surface.^44^ The Nyquist plots of the chitosan-ImH@γ-CD composite coating have a larger semicircular diameter compared to the chitosan (chitosan@Mg) coating, indicating a higher corrosion resistance of the former. This higher resistance can be attributed to the presence of imidazole in the structure of γ-CD MOF. As the immersion time increases, the diameter of the semicircle in the Nyquist plots and the value of |*Z*| increases, while the maximum phase angle gradually decreases and shifts to lower frequencies. This behavior can be caused by the decrease in the absorption of anions such as Cl^−^, HCO_3_^−^, H_2_PO_4_^−^, or HPO_4_^2−^ on the electrode surface and as a result the capacity of its surface layer decreases.^45^ As the immersion time increases, the released imidazole forms a protective layer on the surface of the magnesium alloy, which effectively protects it from corrosion. The parameters obtained from fitting the experimental data with the considered EEC response (inset of Fig. 4C) are presented in Table 2. The values of the *χ*^2^ parameter reported in Table 2 indicate the level of acceptable agreement between the theoretical and experimental data. The reported data also show that the R_ct_ value of the chitosan-ImH@γ-CD composite coating (303.0 Ω cm^2^) is significantly higher compared to the chitosan@Mg coating (169.72 Ω cm^2^). This increase in the resistance is attributed to the inclusion of imidazole into the structure of γ-CD MOF, which leads to an increase in the density and compactness of the coating. As the immersion time increases, the *R*_ct_ value of the chitosan-ImH@γ-CD composite coating increases up to 853.60 Ω cm^2^, which indicates an increase in the corrosion inhibition efficiency of the coating with passage of immersion time. It should be noted that the data obtained from the EIS experiments are in complete agreement with the outcome obtained from the Tafel plots.

**Table 2:** The data obtained from the electrical equivalent circuit (Fig. 4C, inset) used to generate the theoretical plots (Fig. 4C) for the impedance data of the Mg alloy, ImH@Mg, chitosan@Mg and chitosan-ImH@γ-CD composite before and after immersion in SBF for 5 days at 25 °C

Parameters	Sample						
	Before immersion in SBF solution for 5 days				After immersion in SBF solution for 5 days		
	Mg	ImH@Mg	Chitosan@Mg	Composite of chitosan-ImH@γ-CD	Mg	Chitosan@Mg	Composite of chitosan-ImH@γ-CD
*R*_s_ (Ω cm^2^)	0.24	0.32	0.28	0.32	0.26	0.36	0.30
CPE_out_ (Ω^−1^ cm^−2^ s^*n*^)	1.07 × 10^−7^	5.28 × 10^−7^	6.41 × 10^−8^	3.74 × 10^−8^	5.22 × 10^−7^	4.19 × 10^−8^	2.81 × 10^−8^
*n*	1.00	0.87	0.96	1.00	0.85	1.00	1.00
*R*_out_ (Ω cm^2^)	15.00	20.54	32.08	32.72	19.20	34.92	52.08
CPE_in_ (Ω^−1^ cm^−2^ s^*n*^)	5.55 × 10^−5^	5.60 × 10^−5^	3.74 × 10^−5^	2.84 × 10^−4^	2.91 × 10^−5^	5.84 × 10^−4^	5.49 × 10^−4^
*n*	0.79	0.78	0.75	0.56	0.85	0.89	0.85
*R*_in_ (Ω cm^2^)	189	239	657	717	197	895	1140
CPE_ct_ (Ω^−1^ cm^−2^ s^*n*^)	5.94 × 10^−3^	3.79 × 10^−3^	2.03 × 10^−3^	1.90 × 10^−5^	1.12 × 10^−3^	9.11 × 10^−4^	7.8 × 10^−4^
*n*	0.90	0.90	0.97	1.00	0.85	0.93	0.90
*R*_ct_ (Ω cm^2^)	58.18	88.36	169.72	303.00	64.88	512.60	853.60
*L* (henri)	744	1508	2312	2450	1362	4816	5650
*R*_L_ (Ω cm^2^)	34.78	126.02	210.8	318.88	105.2	555.86	763.6
*χ* ^2^ × 10^−4^	4.78	7.14	7.85	5.23	1.06	2.30	9.37
IE%	0	34.15	65.72	80.79	10.32	88.65	93.18

### 3.4. MTT test

**Fig. 5:**
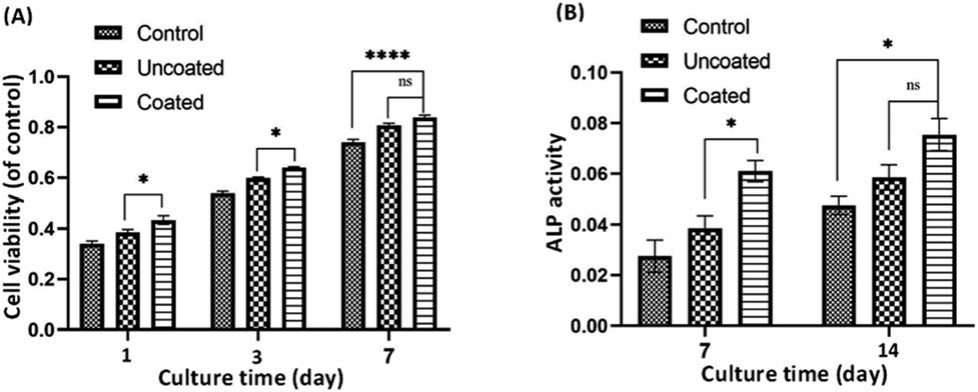
(A) Cell viability of MC3T3-E1 cells grown in culture media containing the extracts of the uncoated and composite of chitosan-ImH@γ-CD-coated Mg alloy for 1, 3 and 7 days. (B) Alkaline phosphatase (ALP) activity of MC3T3-E1 cells treated with uncoated and composite of chitosan-ImH@γ-CD-coated Mg alloy for 7 and 14 days of incubation. (*n* = 3, *p* < 0.05).

The proliferation and survival of MC3T3-E1 osteoblasts to in the extraction media of uncoated and coated Mg alloy were analyzed at 1, 3, and 7 days of culture (Fig. 5A). Cell proliferation was assessed using MTT assay, which measures the activity of mitochondrial dehydrogenases in living cells and correlates it with the number of cells attached to the substrate. The results, averaged over three repetitions (*n* = 3) and reported with a 95% confidence level (*p* < 0.05), demonstrate that the growth of MC3T3-E1 osteoblastic cells increases over time in both the control group and the uncoated and coated Mg alloy groups. Importantly, cell growth in both alloy groups showed a significant enhancement compared to the control group. The uncoated and coated Mg alloy groups exhibited significantly more cell attachment than the controls (*p* ≤ 0.05). However, there was a significant difference (*p* ≤ 0.05) in the proliferation rate between the uncoated and coated Mg alloy during the experimental period. The cell number on the coated Mg alloy was much higher than that on the uncoated Mg alloy. In particular, the cell viability on the composite of chitosan-ImH@γ-CD was significantly higher than that on the uncoated Mg alloy, indicating a favorable effect of the chitosan-ImH@γ-CD composite on MC3T3-E1 cell proliferation. Chitosan has been extensively studied for its diverse applications in the regeneration of various tissues, including skin, cartilage, peripheral nerves, liver, bone, and blood vessels. Research indicates that chitosan significantly enhances the adhesion and proliferation of osteoblasts.^46^ Typically, the growth of osteoblast cells is conducted using DMEM culture medium, which contains a specific concentration of glucose. Consequently, the presence of γ-CD, with its glucose units, may further augment cell proliferation.

### 3.5. ALP activity

Alkaline phosphatase (ALP) is an enzyme marker for the early differentiation of osteoblasts and bone formation and was used to evaluate the level of differentiation of MC3T3-E1 cells in the extraction media of both the uncoated and coated Mg alloy. The intracellular ALP activity was measured after 7 and 14 days of cell incubation as an indication of changes in the differentiation behavior of the bone-forming cells (Fig. 5B). As shown in Fig. 5B, after 7 days of culture, the ALP activity of coated Mg alloy samples was significantly higher than that of uncoated Mg alloy. When the incubation time was extended to 14 days, the expression of intracellular ALP in the coated Mg alloy and uncoated Mg alloy groups increased dramatically, especially in the case of the coated Mg alloy group. This result indicates that the composite of chitosan-ImH@γ-CD effectively enhances the differentiation of MC3T3-E1 cells.

## 4. Conclusions

This study successfully developed a smart pH-sensitive bio-coating by the encapsulating imidazole within cholesterol-modified water-resistant γ-CD MOF. The encapsulation efficiency of ImH is approximately 37.3 mg of ImH per 100 mg of waterproofed γ-CD MOF. The data obtained indicate that, after 60 minutes, the percentages of released imidazole in basic, neutral, and acidic solutions are 97.21%, 30.43%, and 36.35%, respectively. When the magnesium alloy coated with a composite of chitosan-ImH@γ-CD is placed in the SBF solution, the pH of the solution increases near the corrosion site over time. As a result, imidazole is released as a corrosion inhibitor and forms a protective layer on the surface of the alloy, effectively preventing corrosion. Therefore, compared to the chitosan@Mg coating, after 5 days of immersion in the corrosive solution, the composite of chitosan-ImH@γ-CD coating exhibits the highest inhibition efficiency (97.27%), the lowest corrosion rate, and the minimum corrosion current density. Furthermore, the results from the MTT assay show significantly higher cell viability in the composite of chitosan-ImH@γ-CD compared to the bare magnesium alloy, indicating the favorable effect of the composite on the proliferation of MC3T3-E1 cells. Additionally, the ALP results demonstrate that the composite of chitosan-ImH@γ-CD effectively enhances the differentiation of MC3T3-E1 cells.

## Data availability

The data that support the findings of this study are available from the corresponding author upon reasonable request.

## Conflicts of interest

The authors declare that they have no known competing financial interests or personal relationships that could have appeared to influence the work reported in this paper.
